# Integrative transcriptomic analysis of Korean high-grade serous ovarian cancer

**DOI:** 10.1371/journal.pgen.1011660

**Published:** 2025-09-15

**Authors:** Hyondeog Kim, Youngwook Kim, Juyeong Park, Dong-Min Shin, Ji Hyun Kim, Madhawa Gunathilake, So Young Kwon, Junyoung Shin, Chong Woo Yoo, Sang-Yoon Park, Myong Cheol Lim, Jeongseon Kim

**Affiliations:** 1 Graduate School of Cancer Science and Policy, National Cancer Center, Goyang, Gyeonggi-do, Republic of Korea; 2 Department of Medicine, Yonsei University College of Medicine, Seoul, Republic of Korea; 3 Theragen Bio Co., Ltd, Seongnam, Gyeonggi-do, Republic of Korea; 4 Center for Gynecologic Cancer, National Cancer Center, Goyang, Gyeonggi-do, Republic of Korea; 5 Division of Cancer Data Science, Research Institute, National Cancer Center, Goyang, Gyeonggi-do, Republic of Korea; 6 Department of Pathology, National Cancer Center, Goyang, Gyeonggi-do, Republic of Korea; 7 Center for Clinical Trial, National Cancer Center, Goyang, Gyeonggi-do, Republic of Korea; 8 Division of Rare and Refractory Cancer, Research Institute, National Cancer Center, Goyang, Gyeonggi-do, Republic of Korea; 9 Department of Cancer Control and Policy, National Cancer Center, Goyang, Gyeonggi-do, Republic of Korea; Huazhong University of Science and Technology Tongji Medical College, CHINA

## Abstract

High-grade serous ovarian cancer (HGSOC) is the predominant subtype of ovarian cancer and is characterized by a high rate of relapse after platinum-based chemotherapy. Herein, we present a comprehensive analysis of 111 Korean HGSOC samples using next-generation sequencing technology to elucidate their transcriptomic landscapes. Our investigation revealed the existence of four distinct transcriptional subtypes of ovarian cancer: immunoreactive, mesenchymal, proliferative, and differentiated, which is comparable to those of TCGA HGSOC transcriptional subgroups. Each subtype exhibited unique correlation networks and their immune cell composition was computationally determined. Notably, the immunoreactive cluster displayed the highest immune score, even in the context of pan solid-cancer types, accompanied by heightened expression of CD4^+^ and CD8^+^ T cells (**P* *< 0.05), along with notable associations with neutrophil degranulation and antigen presentation pathways (FDR < 0.01). Conversely, the differentiated cluster demonstrated immunodepleted characteristics, featuring an elevated proportion of overexpressed cancer-germline antigens. We also identified several cancer-germline HGSOC antigens that could be further investigated as potential targets for immunological intervention in cancer.

## Author summary

Our study presents an extensive investigation of the transcriptomic landscape of high-grade serous ovarian cancer (HGSOC) in an East Asian population. Our analysis identified an immunoreactive subgroup within the HGSOC population that displayed remarkably heightened immune activity, presenting a promising avenue for immune-based therapies for this challenging disease. Additionally, we identified cancer-germline antigens as potential targets for innovative immuno-oncological interventions to address critical clinical needs. Moreover, our study established a highly accurate predictive model that integrates transcriptomic markers with clinical factors, offering valuable insights for personalized treatment strategies. These findings contribute to a broader understanding of the pathogenesis of HGSOC and pave the way for novel therapeutic approaches with the potential to improve patient outcomes.

## Introduction

Ovarian cancer is one of the most common gynecological malignancies globally and is a significant cause of morbidity and mortality, accounting for 207,252 new deaths each year [1]. The 5-year survival rate of this cancer is reported to be 26–42% and it is often diagnosed at an advanced stage [2]. Epithelial ovarian cancer is a heterogeneous disease with several histological subtypes. High-grade serous ovarian cancer (HGSOC) is the most common cancer subtype (70–80%) and it is highly aggressive and grows rapidly [3].

Despite the initial success of cytoreductive surgery and platinum-based chemotherapy, over 75% of patients with HGSOC experience relapse after completion of first-line therapy [4,5]. The challenge of tailoring therapeutic interventions to control progressive disease is compounded by the inherent cellular heterogeneity and genomic instability of HGSOC [5]. While platinum chemotherapy remains the cornerstone of contemporary treatment, the grim reality persists that the majority of patients with epithelial ovarian cancer develop chemotherapy resistance, resulting in a five-year survival rate of less than 50% [6].

Numerous ovarian cancer studies have underscored the prognostic significance of factors such as age at diagnosis, disease stage, grade, histology, residual disease after surgery, and disease recurrence [5,7]. The genetic predisposition towards epithelial ovarian cancer indicates that HGSOC harbors high frequency of germline mutations in *BRCA1/2* [3] as well as significant chromosomal instability with somatic mutations in tumor protein 53 (*TP53*) gene. Molecular markers such as *BRCA1/2* mutations and homologous recombination DNA repair deficiency (HRD) in HGSOC have been validated as predictors of the response to platinum therapy and poly (ADP-ribose) polymerase (PARP) inhibitors [8–10]. Recent research has shed light on the prognostic potential of immune cell infiltration in ovarian tumor tissues [11].

The Cancer Genome Atlas (TCGA) and other genomic studies on HGSOC have conducted extensive genomic and transcriptomic analyses to delineate its genomic landscape and facilitate the development of targeted therapies for this highly lethal malignancy [3,12,13]. Key findings from previous studies include the prevalence of *TP53* gene mutations; the frequent and widespread occurrence of DNA copy number alterations; and the identification of transcriptional signatures associated with clinical outcomes and molecular subtypes, and of diverse mechanisms of *BRCA1/2* inactivation [3,12,14]. However, most studies have focused on HGSOC in white populations, whereas there is limited comprehensive molecular characterization of HGSOC in the East Asian population, including that in Korea [15–17].

In this study, we aimed to identify the clinical and molecular factors that distinguish 111 patients with HGSOC from the Korean population through an integrated analysis of clinical features, tumor transcriptomic data and their tumor immune microenvironment.

## Results

### Clinicopathological characteristics of the study participants

A total of 111 HGSOC cases were analyzed in this study. The clinicopathological characteristics, including age, tumor grade, tumor stage, postoperative residual disease, and overall survival, are summarized in Table 1. The mean age of the patients was 54 years. Most patients had tumors of histological grade 2 and 3 (94.7%) and FIGO stage III (51.8%). As postoperative residual disease, almost 79.5% of patients had microscopic disease, while 18.8% and 1.8% showed < 1 cm and ≥ 1 cm residual disease, respectively (Tables 1 and S1).

**Table 1:** Clinicopathological characteristics of 111 high-grade serous ovarian cancer.

Patient characteristics	n (%)
Age at diagnosis± SD	54.1 ± 8.6
Tumor grade	
G1	5 (4.5%)
G2	44 (39.6%)
G3	62 (55.9%)
FIGO stage	
I	1 (0.9%)
II	6 (5.4%)
III	58 (52.3%)
IV	46 (41.4%)
Neoadjuvant chemotherapy	
Yes	40 (36.0%)
No	71 (64.0%)
Postoperative residual disease	
Microscopic	88 (79.3%)
<1 cm	21 (18.9%)
≥1 cm	2 (1.8%)
Vital status	
Alive	55 (49.5%)
Death	12 (10.8%)
LFU	44 (39.6%)
Mean overall survival in years ± SD	3.90 ± 1.69

FIGO: The International Federation of Gynecology and Obstetrics; G: grade; LFU: lost to follow-up; SD: standard deviation

### Somatic alterations of HGSOC

**Fig 1:**
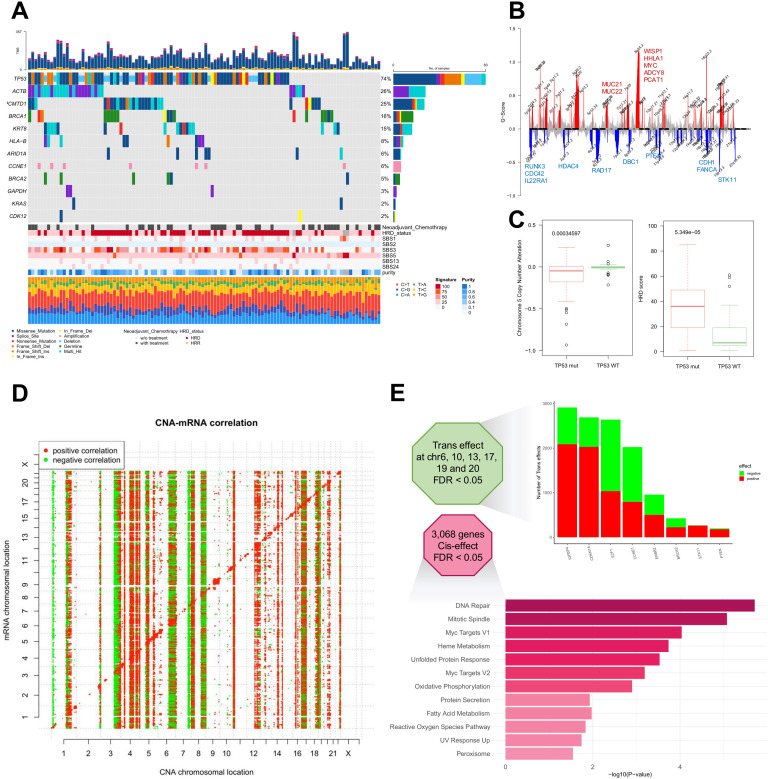
Genomic landscape of 111 high grade serous ovarian cancer Korean patients. **(A)** Somatic mutational profile of 11 frequently mutated genes along with copy number variation. Shown above the oncoplot is the tumor mutational burden. Shown below is the clinical status of neoadjuvant chemotherapy, homologous recombination deficiency (HRD) status inferred from exome sequencing data and mutational signatures operative in the cohort. **(B)** The copy number alteration (CNA)-mRNA correlation graph illustrates positive correlation depicted in red and negative correlation depicted in green (FDR < 0.05, top). CNA-driven cis effects manifest as red diagonal lines, while trans effects are discernible as vertical lines alternating between red and green. Copy number alteration over all chromosomal location figure with G-score depicting the significance (bottom). Genes covered by significant altered chromosomal locations are labelled. **(C)** boxplot depicting significant difference in chromosome 5 CNA and HRD score between *TP53* mutated group against *TP53* wild type group. **(D)** Bar plot visualizes the number of the trans effect caused by genes on x-axis, red as positive and green as negative correlation (FDR < 0.05, top). Gene set enrichment analysis of 3,068 genes which have cis-effect with positive correlation (bottom).

To elucidate the genomic features associated with HGSOC in an East Asian population, we conducted exome and transcriptome analyses of 111 primary tumors along with healthy matched tissue from patients who passed the pathological review and molecular quality control criteria for subsequent parallel sequencing. Exomes were sequenced with a median coverage of 134× for both tumors and normal samples. The tumor transcriptomes were sequenced, yielding a median of 63 million reads per sample. Our analysis revealed a median mutation frequency of 1.24 non-synonymous mutations per megabase (Fig 1A) (range 0.10–5.26), which is comparable to the frequency found in the TCGA dataset (median of 0.86 mutations per megabase). Patients with high tumor mutational burden (TMB) underwent mutational signature analysis to assess microsatellite instability (MSI). The high TMB samples were primarily associated with either SBS1, SBS5, or SBS3 signatures, representing clock-like or HRD signatures, respectively (Fig 1A). MSI-related signatures (SBS6, SBS14, SBS15, SBS20, SBS21, SBS26, and SBS44) were largely absent, with only SBS6 observed at a minimal level within the cohort.

In line with previous genome landscape studies on HGSOC, *TP53* (83/111, 74%), *BRCA1* (18/111, 16%), and *BRCA2* (6/111, 5%) were some of the most commonly mutated genes in our cohort (Fig 1A). We compared tumor purity between patients with mutated and wild-type *TP53* and found no discernible differences in the distribution of tumor purity levels (S1A Fig). Moreover, comparable proportions of patients with relatively low purity were observed in both the *TP53* mutant and wild-type groups, indicating that the absence of *TP53* mutation was unlikely to result from technical artifacts. *MDM2* gene amplification status did not differ between patients with mutated and wild-type *TP53*. Additionally, our cohort exhibited a 6% frequency of copy number alterations in *CCNE1*, primarily through amplification. Less frequently mutated genes in HGSOC (*GAPDH*, *KRAS* and *CDK12*) were found in less than 10% of our cohort, which is consistent with the findings from the TCGA dataset. Mutational signature analysis revealed the presence of the SBS3 signature across our samples, indicating defective repair of double-strand DNA breaks due to homologous recombination deficiency (HRD) (Fig 1A).

Statistically significant recurrent focal somatic copy number alterations were also observed in other chromosomal regions, including significantly amplified 8q24 region, encoding *MYC* and 19q13, encompassing *AKT2* (Figs 1B and S1B and S2 Table). Significant deletions were observed in regions such as 5q11-13, frequently reported as loss of heterozygosity (LOH) in ovarian cancer [15], 8p23 encoding *DBC1* (deleted in breast cancer 1 gene), 10p24 harboring *PTEN*, 16q24 encoding *CDH1* (E-cadherin) and *FANCA*, and 19q13.3 encompassing *STK11* (LKB1).

The current ovarian cancer cohort was associated with frequent LOH on chromosome 5, which coincided with LOH on chromosome 5p and *TP53* mutations (Fig 1C, left panel). *TP53*-mutant samples also exhibited higher levels of genomic HRD (Fig 1C, right panel).

To understand the impact of copy number alterations (CNAs) on mRNA abundance, we analyzed their overall cis-effects and trans-effects. Cis-effects represent the direct influence of genetic copy number changes on gene expression within the same chromosomal region, where an increase or decrease in gene copies directly correlates with proportional changes in mRNA levels. Trans-effects, in contrast, reveal a more intricate regulatory process, where copy number changes in one chromosomal location can indirectly modulate gene expression across different chromosomes through complex cellular signaling networks, transcriptional regulatory mechanisms, and intricate molecular interactions, as previously described [18] (Fig 1D). A total of 2,790 genes exhibited a significant positive correlation between CNA and the transcriptome (FDR < 0.05, S3 Table). Gene set enrichment analysis was conducted to identify the genes that were positively influenced, revealing enrichment in pathways associated with cell proliferation, such as Myc targets, DNA repair, and mitotic spindles (Fig 1E). Additionally, pathways, such as the unfolded protein response and metabolic pathways, were found to be under cis-copy number control. Genes with the most widespread impact on the expression of other genes were predominantly located on chromosomes 6, 10, 13, 17, 19, and 20 (FDR < 0.05). Notably, *IGFBP4* emerged as the most influential gene, positively regulating 2,158 genes and negatively regulating 831.

### Molecular clustering of the transcriptome data

**Fig 2:**
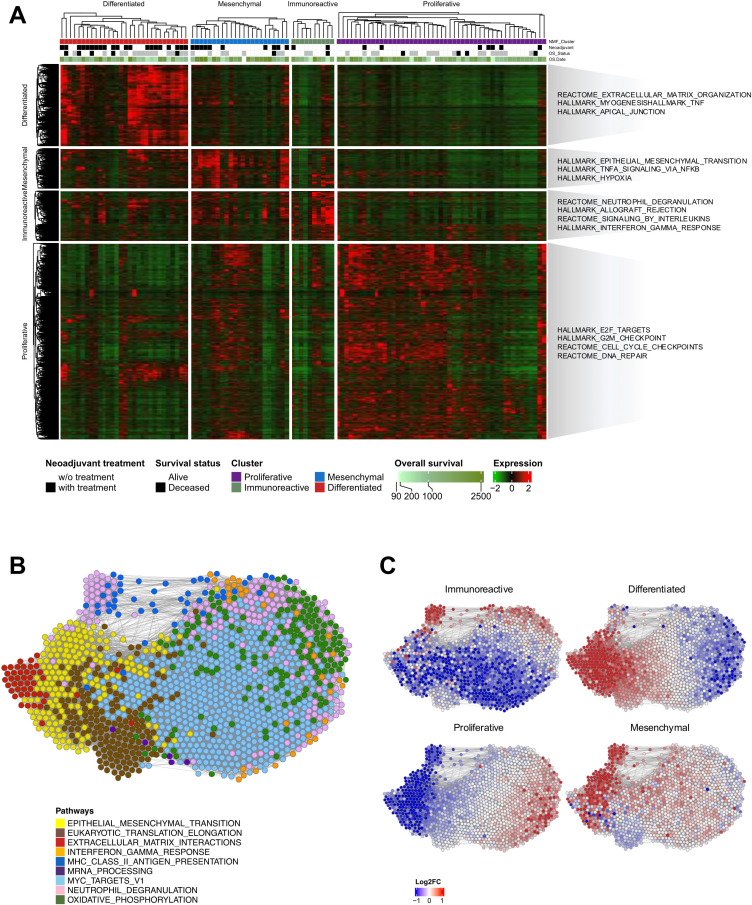
Molecular clustering of transcriptomic data from HGSOC samples. **(A)** A total of 4,597 genes were selected after filtering by median absolute deviation and four clusters were grouped by non-negative matrix factorization (NMF) clustering. Clusters were identified using transcriptomic features that were exclusive to each cluster, and enriched pathways are annotated on the right. **(B)** Gene correlation network analysis using 1,949 genes that have correlations with each other. Ten pathways found to be enriched were annotated with different colors. **(C)** The gene correlation network colored with log-transformed fold change of each cluster compared to the others: red represents higher expression in the corresponding cluster and blue color lower expression.

HGSOC were further explored using transcriptomic clustering. A total of 111 HGSOC samples were grouped into four non-negative matrix factorization (NMF) clusters based on pathway enrichment analysis: immunoreactive, proliferative, mesenchymal, and differentiated (Figs 2A and S2A and S4 Table). This clustering was consistent with previous findings in the TCGA cohort [18]. Notably, the differentiated and mesenchymal clusters predominantly included patients who had undergone neoadjuvant treatment, suggesting the influence of this treatment on the associated genes. Upon subsetting our cohort based on neoadjuvant treatment history, we observed the absence of differentiated clusters in 40 treated samples, indicating the rapid depletion of markers associated with tumor differentiation following neoadjuvant chemotherapy in bulk tumors (S2B and S3A Figs). We analyzed 72 treatment-naïve samples and designated four NMF clusters, annotated as mesenchymal, differentiated, cytopathies, and immunoreactive (S2C and S3B Figs).

Analysis of the gene-to-gene correlation network identified 1,895 genes that exhibited significant correlations with other genes. Nine pathways with statistically significant correlations were identified through this investigation (Fig 2B). Furthermore, by assessing the fold change in gene expression specific to each molecular subtype, we delineated distinct characteristics within each cluster (Fig 2C). The immunoreactive cluster was distinguished by its association with neutrophil degranulation and MHC class 2 antigen processing and presentation pathway. The mesenchymal clusters exhibited overall upregulated characteristics, with the most prominent features being indicative of epithelial–mesenchymal transition and neutrophil degranulation. The differentiated clusters exhibited prominent characteristics related to extracellular matrix interaction and eukaryotic translational elongation. Finally, the proliferative cluster showed attributes linked to Myc targets and oxidative phosphorylation.

High expression of transcription factors, such as HMGA2 and proliferation markers, such as MCM2 and PCNA defined the proliferative subtype. The differentiated subtype was associated with high expression of ovarian tumor markers (MUC1 and MUC16) and the secretory fallopian tube marker SLPI, suggesting a more mature stage of development. High expression of epithelial–mesenchymal transition markers such as VIM and microvascular pericytes (ANGPTL2 and ANGPTL1) characterizes the mesenchymal subtype. High expression levels of CD44, CD74 and the chemokine C–C motif ligand (CCL) represented an immunoreactive cluster.

### Computationally measured immune cell composition reveals characteristics of the HGSOC clusters

**Fig 3:**
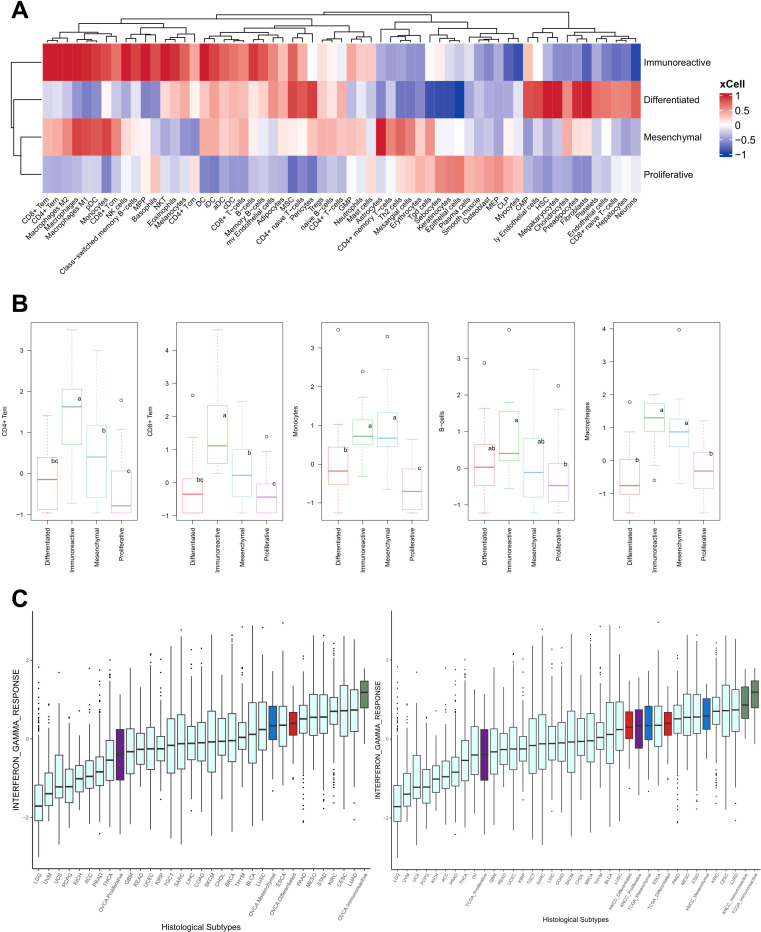
Analysis of immune composition by clusters. **(A)** Heatmap representation of immune deconvolution using xCell shows the four NMF RNA molecular subgroups. The x-axis shows different deconvoluted immune cell types and y-axis correspond to different molecular subgroups. **(B)** Boxplots exhibiting significant difference of immune cell enrichment of each cluster. Statistical analysis was conducted using analysis of variance (ANOVA). Lowercase letters (a, b, c) in the boxes indicate groupings of means, where groups with different letters are significantly different (**P* *< 0.05) based on post hoc tests. **(C)** Comparison of overall enrichment of the immune related pathways in 29 TCGA pan cancer subtypes integrated with the KNCC-OV cohort, highlighting when decomposed by the TCGA cluster and 4 KNCC NMF clusters, respectively.

We applied computational deconvolution algorithms to the bulk transcriptome data (Fig 3A) to estimate immune cell composition in the tumor microenvironment (Figs 3A and S4A). Consistent with the gene correlation network analysis, immunoreactive clusters exhibited significantly higher immune scores than the other clusters (Fig 3B, *P < 0.01*, analysis of variance, ANOVA). This cluster demonstrated a marked enrichment of CD8^+^, CD4^+^ T cell subsets, and monocytic cells compared to the other clusters (Fig 2B). The differentiated cluster exhibited the highest proportion of fibroblasts (S4B Fig) and the proliferative subgroup comprised heterogeneous cell types, including epithelial, smooth muscle, and osteoblastic cells (S4C Fig).

To assess the immune tumor microenvironment of ovarian cancer compared to that of other cancer types, we conducted a comparative analysis of the immune tumor microenvironment in HGSOC against other cancer types. Initially, by leveraging the TCGA pan-cancer dataset, we compared the immune scores of TCGA-HGSOC RNA subgroups with those of other TCGA cancer types. Notably, the immune gene set enrichment, such as the interferon γ response genes, in the TCGA-HGSOC immunoreactive cluster stood out prominently within the pan-cancer context (Fig 3C, left panel). This heightened immune score within the immunoreactive subgroup of HGSOC was externally validated by analyzing our own HGSOC samples after batch correction and merging them with the TCGA pan-cancer data. The immune landscape of the pan-cancer cohort merged with our own data in a consistent fashion, validating that the immune microenvironment of immunoreactive HGSOC had the highest immune activity (right panel of Figs 3C and S5A).

Subsequently, we explored the potential correlation between the immune score of HGSOC and other molecular markers with prognostic implications. When the immune score of HGSOC was compared with the measure of homologous recombination deficiency, no discernible correlation was observed (S5B Fig). This suggests that while immune cell infiltration and homologous recombination deficiency each possess prognostic significance in HGSOC, they operate as largely orthogonal and independent factors contributing to the clinical outcome of HGSOC.

### Gene dependency and vulnerability analysis

We utilized data from CCLE and DepMap to pinpoint vulnerabilities and potential treatment avenues in ovarian cancer subgroups. Our preliminary investigation focused on 21 HGSOC cell lines that were accessible from a public database. Compared with other cancer cell lines, ovarian cancer cells displayed a notable dependency on *TOR2A* and integrin-linked kinase (S6A Fig and S5 Table).

NMF clustering and pathway enrichment were performed and the cell lines were subsequently grouped into four distinct subgroups using the same algorithm applied to the Korean National Cancer Center (KNCC) ovarian cancer cohort. To identify subgroup-specific targets, we investigated genes that had a significant impact on subgroup perturbation. The immunoreactive cluster exhibited dominantly high enrichment in estrogen response pathways followed by interleukin signaling and inflammatory response. This revealed that the immunoreactive subgroup exhibited a heightened dependency on the *IPPK* and *WHAMM* gene compared with the other groups (**P* *= 0.01 and *P* = 0.001, respectively, S6B Fig). Cell lines in the mesenchymal subgroup showed a strong dependency on *GPI* (**P* *= 0.005, S6C Fig), whereas those in the proliferative subgroup demonstrated a pronounced dependency on *ACSL3, XPOT* and *IRS2* (**P* *< 0.0005, S6D Fig). Cell lines belonging to the differentiated subgroup exhibited notable reliance on *CKS1B* (**P* *= 0.009, S6E Fig).

### Cancer-germline antigen as a potential immunological target

**Fig 4:**
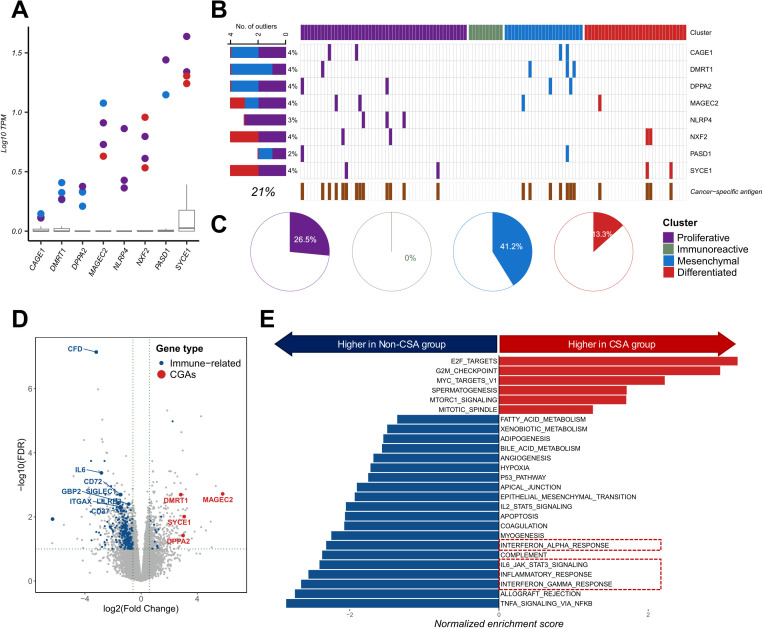
Candidate HGSOC-specific antigen distribution and proportion in the cohort. **(A)** Boxplot exhibiting log10 TPM value of heightened expression of cancer-germline antigens (CGAs). **(B)** Heatmap showing cancer-germline antigen distribution by cluster. The x-axis represents samples and y-axis shows candidate HGSPC-specific antigens. **(C)** Proportion of CGA overexpression samples by clusters. **(D)** Differentially expressed genes with threshold of FDR < 0.01 and log2 fold change over 1.5. Significantly different immune-related genes are colored in blue whereas significantly different CGAs are colored and labelled in red. **(E)** Gene set enrichment score comparison of the CGA overexpressed group against the other group.

Cancer-germline antigens are exclusively present in cancer cells and germline tissues and have thus been consistently pursued as promising targets for cancer immunotherapy, owing to their potential to enhance treatment efficacy while minimizing unwanted toxicity to normal cells. To identify patients who were most likely to have cancer-germline antigens, we examined the transcriptome data and identified genes that had at least one sample with significantly higher expression than other samples (Fig 4A and Methods and S6 Table). These genes were further screened for expression confined to normal germline tissues (testes and ovaries) using data from the GTEx project (S7 Fig).

In our cohort, 21% of the samples exhibited atypically elevated cancer-germline antigen expression and were mostly clustered in the non-immune subgroups (Fig 4B). The prevalence of cancer-germline antigen overexpression varied among subtypes (Fig 4C). The mesenchymal subgroup showed the highest proportion of cancer-germline antigen overexpression samples (41.2%), followed by the proliferative cluster (26.5%) and differentiated subgroup (13.3%). In contrast, the immunoreactive subgroup did not exhibit cancer-germline antigen overexpression.

To gain deeper insights into the molecular distinctions associated with the overexpression of cancer-germline antigens, we conducted differentially expressed gene analysis between patients with and without cancer-germline antigen expression. Downregulated genes in the cancer-germline antigen-containing group were mainly immune-related genes and pathways, such as interleukin family signaling and the interferon response (Fig 4D and 4E).

### Prediction of overall survival using clinical and transcriptomic factors

**Fig 5:**
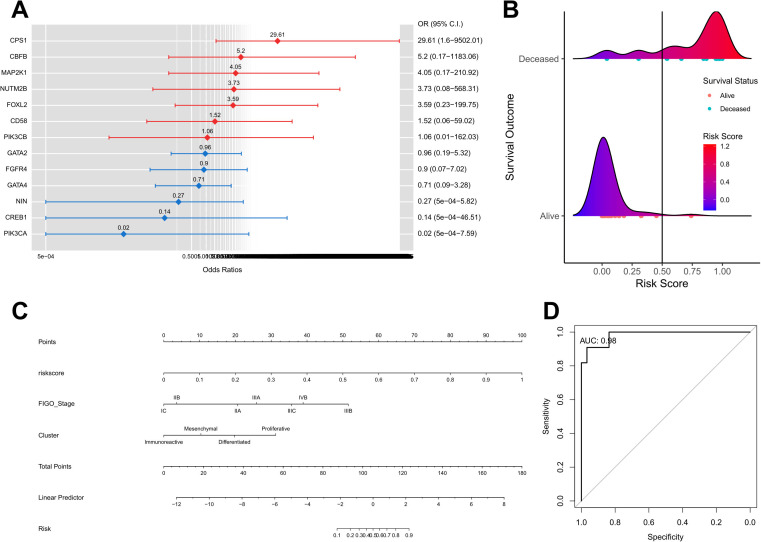
Construction of nomogram predicting overall survival of KNCC-HGSOC cohort. **(A)** Logistics regression analyses of transcriptomic factors associated with overall survival. **(B)** Ridgeline plot that visualizes the density of the distribution of risk score by survival status. **(C)** The nomogram consists of grade, neoadjuvant treatment status, transcriptomic NMF-clusters, and the risk score based on the regression coefficients. **(D)** ROC curves according to the nomogram and transcriptomic risk score.

The prediction of the clinical prognosis for HGSOC commenced with gene filtration using a logistic regression model (Fig 5A). Subsequently, the risk scores were computed for each sample based on the obtained coefficients. Mapping the risk scores by survival status revealed a higher density of distribution among the surviving groups when the risk score was low (Fig 5B). Following this, a nomogram was developed incorporating clinical factors, such as FIGO stage, NMF clusters, and risk score (Fig 5C). The nomogram aids in predicting overall survival probability by summing the points assigned to each independent covariate and locating the corresponding total points on the scale. The predictive performance of the nomogram was validated using a receiver operating characteristic (ROC) curve, yielding an AUC score of 0.98 (Fig 5D).

## Discussion

Our investigation focused on patients with HGSOC aged 20 years and older and involved integrative transcriptomic analyses. To the best of our knowledge, this is the largest concurrent clinico-genomic cohort of patients with HGSOC from East Asia, providing novel insights into the immunological landscape of HGSOC within the pan-cancer framework. It also presents a list of candidate cancer-germline antigens for this disease, which is characterized by significant unmet clinical needs.

We observed a highly heterogeneous immune activity in the HGSOC cohort. Samples classified into the immunoreactive subgroup exhibited the highest levels of immune activity compared to all other TCGA pan-solid cancer types, whereas certain samples in the non-immune subgroup displayed one of the lowest levels of immune activity within the pan-solid cancer context. The notably increased immune activity observed in the immunoreactive subtype of HGSOC suggests that patients within this cluster may exhibit a high response rate to appropriate oncological interventions. Even in a broader pan-cancer context, the immune activity of the immunoreactive subgroup of HGSOC was remarkably elevated, featuring notable expression of interferon response genes and other immune gene markers. Interferon gamma (IFN-γ) response in ovarian cancer is a topic of interest due to its potential role in the tumor microenvironment and immune modulation. Studies have shown that IFN-γ signaling pathways can exert both anti-tumor and pro-tumor effects, including tumor growth, metastasis, and immune evasion in ovarian cancer [19]. On one hand, IFN-γ has been shown to inhibit ovarian cancer cell proliferation and induce apoptosis, suggesting its potential as a therapeutic agent. On the other hand, IFN-γ enhances the anti-tumor immune response by activating cytotoxicity in immune cells such as NK and CD8^+^ T cells, and combining IFNs with modalities such as immunotherapy or TLR4 agonists show promise in reversing immunosuppression and boosting cytotoxicity against ovarian cancer cells, offering effective therapeutic approaches for combating the disease [20]. However, the relationship between IFN-γ and ovarian cancer is complex, as IFN-γ signaling can also promote tumor progression and immune escape mechanisms. For example, IFN-γ has been implicated in the induction of immunosuppressive factors such as indoleamine 2,3-dioxygenase and programmed death-ligand 1*,* which can inhibit T cell function and promote immune tolerance within the tumor microenvironment [20,21]. Further research is needed to elucidate the precise mechanisms underlying the modulation of immune activity in ovarian cancer and determine its therapeutic potential in clinical settings. Increased lymphocytic infiltration into the tumor microenvironment is a histological feature frequently observed in HGSOC. The correlation between infiltrating immune cells and patient survival depends on both the composition and number of cell types present. Specifically, CD4^+^ T, CD8^+^ T, and B cells have been linked to enhanced clinical outcomes, whereas regulatory cell types, such as regulatory T cells and neutrophils, have been associated with poorer outcomes in ovarian cancers [22]. Recent immuno-oncological approaches have prioritized enhancing the cancer-immunity cycle by modulating the activity of infiltrating immune cells with tumor-killing capabilities and increasing the access of these lymphocytes to cancer cells. Using whole-transcriptome analysis and publicly available gene sets, we inferred the enrichment of lymphocytic infiltration as a whole and the presence of individual subtypes of immune cells in each patient with HGSOC. This approach confirmed that the immunoreactive subtype of HGSOC correlated with the immune score determined by immune cell deconvolution methods and the decomposed immune components that were directly linked to a heightened immune landscape. We conducted DEG and GSEA analyses using RNA and CNA (S8A and S8B Fig). The *TP53* mutated group demonstrated high enrichment scores in GSEA for cell proliferation-related pathways, including E2F targets and Myc targets, and predominantly comprised the proliferative group. Furthermore, differential expression analysis between *TP53* mutated and wild-type groups based on copy number revealed multiple oncogenes in the *TP53* mutated group. These findings suggest that the *TP53* mutated group may exhibit more aggressive characteristics.

Using transcriptome data, we identified a set of genes whose expression was specifically enriched in bulk tumor tissues compared to normal expression data from public atlases, potentially identifying these genes as cancer-germline antigens in HGSOC. Notably, outlier patients with high expression of these cancer-germline antigens mostly clustered into the non-immune subtypes. Targeting cancer-germline antigens offers advantages because they are typically expressed exclusively in cancer cells, thereby minimizing the risk of off-target side effects and reducing damage to healthy cells. Furthermore, selected cancer-germline antigens are almost exclusively expressed in testis cells, which dramatically reduces the likelihood of causing damage to normal cells when targeted, considering the characteristics of ovarian cancer exclusively occurring in biological females. The differentiated and proliferative clusters, which are the two groups with the highest proportion of cancer-germline antigens within the group, exhibited immune depletion with a high stromal cell presence, which may be associated with a high rate of immune evasion occurring in these clusters [23]. We found that *HMGA2* as highly expressed transcription factor which is consistent with the findings reported by Wu J et al. [24] where *HMGA2* is correlated with aggressiveness of HGSOC. Moreover, in proliferative subtype, higher expression of certain proliferation markers such as *MCM2* and *PCNA* and in differentiated subtype, higher expression of *MUC1*, *MUC16* and *SLP1* also were consistent compared to previous studies [25–28]. In contrast, Garsed GW et al. [29] reported that *BRIP1*, *PALB2*, and *RAD51C* as frequent germline mutations whereas *ATM*, *CDK12*, *PTEN*, *RAD51B*, *RAD51C*, and *RAD51D* as frequent somatic mutations in HGSOC.

In this study, we identified prognostic markers to predict clinically relevant differences in overall survival. *MAP2K1* was found to have the top 3 highest hazard ratio. *MAP2K1* encodes a mitogen-activated protein kinase (MAPK), which exhibits activating mutations implicated in several cancers including ovarian, melanoma, and lung cancers, and its inhibition has demonstrated efficacy in restraining tumor growth in these contexts [30]. Furthermore, previous studies have analyzed large-scale predictive markers with 3,769 HGSOC samples from six publicly available databases, and the identified markers were strongly enriched in the phosphatidylinositol 3-kinase and MAPK pathways [31]. The MAPK and phosphatidylinositol 3-kinase pathways are controlled by receptor tyrosine kinases and are interconnected, often working in concert to regulate cellular responses to various extracellular signals [32]. This consistent pattern underscores the crucial role of the crosstalk and coordination among receptor tyrosine kinases in preserving ovarian cancer homeostasis and resilience to alterations in the cellular environment during treatment.

This study had several limitations that warrant further investigation. In subsequent analyses, systematic long-term follow-up of patients’ clinical data is needed to enhance the efficacy of associating molecular factors with clinical elements. Presently, our aim was to longitudinally track the clinical course of this cohort over a period of 2 years, enabling future research endeavors to incorporate aspects of clinico-genomic integrative analyses. Additionally, although there have been several external statistical genomic validations in other cohorts, this study lacks experimental and functional validation, a deficiency that should be addressed in future functional genomic research endeavors through careful clinical design.

In summary, these findings lay the groundwork for developing therapeutic strategies targeting aberrant genes or networks in HGSOC in an East Asian cohort. These approaches involve identifying and precisely targeting specific aberrations using tailored therapies aimed at efficacy. Combined with the future systematic annotation of clinical data in the cohort and other molecular data layers, this merits further investigation into the biology of HGSOC in the context of drug treatment and precision medicine.

## Materials and methods

### Ethics statement

This study was conducted in accordance with the ethical guidelines of the National Cancer Centre’s Institutional Review Board. This study was approved by the National Cancer Center Institutional Review Board (approval number: NCC2022–0264), and written informed consent was obtained from all participants prior to their involvement in the study.

### Study population and sample collection

In this study, Korean women aged > 20 years who underwent surgery at the National Cancer Center for epithelial ovarian cancer between January 1, 2013, and June 22, 2022, were recruited as eligible study participants. Those participants, who provided tissue and blood stored in the National Cancer Center Biobank were recruited. In total, 123 patients provided their biological samples. We have undergone two rounds of independent pathology review to confirm HGSOC pathology and have removed 12 samples from the analysis. Finally, 111 HGSOC patients were included in the analysis (S1 Table). Each eligible study participant provided consent for the donation of human specimens for research purposes prior to surgery. All tumor samples were registered at the National Cancer Center Biobank. Tumor tissue samples were obtained from ovarian or fallopian tube. Tumor tissues were stored as fresh-frozen samples immediately after resection for sequencing. Independent pathological review of the tumor samples was carried out by specialized two pathologists in gynecology for histological diagnosis of all samples. Sequencing of the entire cohort was done on fresh samples. Since HGSOC surgery often involves multiple sites whose respective tumor profile is heterogeneous, we have not included pathological tumor content data.

### Whole exome sequencing data processing

Whole exome sequencing was performed, and initial data quality control was performed using Cutadapt v.4.1 [33] to trim adapter sequences and bases below a quality score of Q20. The cleaned reads were aligned to the human reference genome GRCh37 using Burrows–Wheeler Aligner (BWA) v.0.7.17 [34]. Duplicates were marked using the Genome Analysis Toolkit (GATK) v.4.3 [35]. MarkDuplicates tool, followed by base recalibration using the BaseRecalibrator and ApplyBQSR tools within the GATK.

### Variant calling and oncoplot analysis

Somatic and germline variants were detected separately. Somatic variants and short indels were identified using Mutect2 v.4.3, and only those that passed the Filter PASS criteria were included in the somatic onco-plot. Germline variants were called using HaplotypeCaller v.4.3 [33,34,36]. Only germline variants categorized as pathogenic or likely pathogenic according to the ClinVar database [35] were included in the germline onco-plot [37]. The oncoplots differentiated copy number variations, labeling those with a copy number greater than 4 and less than 1 as “Amp” and “Del,” respectively.

### Copy number variation and homologous recombination deficiency analysis

Copy number variations were analyzed using Sequenza [38] and a CNV kit [39], considering ploidy. GISTIC analysis was conducted to identify regions with significant amplification and loss (q < 0.01). Allele-specific copy number segment files were created using the Sequenza software. Segments were assigned to one of the six types of LOH: heterozygous (HET), neutral LOH (NLOH), deletion LOH (DLOH), balanced copy number amplification (BCNA), amplification LOH (ALOH), and ASCN amplification (ASCNA). The HRD score is measured by combining genomic instability indices, such as LOH, telomeric allelic imbalance (TAI), and large-scale state transitions. Samples were annotated as “HRD” if their total HRD score was higher than 25, which was the median; samples with lower values were annotated as “HRR.”

### Copy number alteration analysis

The significance of CNAs was calculated based on the G-score, which represents the degree of amplification or deletion of a genomic region relative to the background noise from GISTIC analysis [40]. A positive G-score indicated amplification, whereas a negative G-score indicated deletion. Regions with high G-scores are considered to harbor potentially significant genomic alterations that may play a role in cancer development and progression (S2 Table).

The comprehensive impact of CNAs on genome-wide gene expression was investigated using a significance threshold of FDR < 0.05. In total, 2,790 genes exhibited significant cis-effects on the transcriptome, with 2,767 genes displaying a positive correlation and 23 genes showing a negative correlation. The genes exhibiting a positive correlation were subsequently subjected to gene set enrichment analysis using the “KEGG_2021_Human” databases.

### Mutation signature analysis

Mutation signature profiling was conducted using SigProfiler Extractor [38,41]. SBS signatures were extracted from the COSMIC database [39]. These data were visualized along with the clinical information using a heat map.

### RNA sequencing and data processing

The adapter sequences and ends of the reads with a Phred quality score less than 20 were trimmed, and reads shorter than 50 bp were removed simultaneously using cutadapt v.2.8 [36]. Filtered reads were mapped to the reference genome related to the species using the aligner STAR v.2.7.1a [42] following ENCODE standard options (refer to “Alignment” of “Help” section in the html report) with “-quantMode TranscriptomeSAM” option for estimation of transcriptome expression level. Gene expression was estimated using RSEM v.1.3.1 [43] considering the direction of the reads corresponding to the library protocol using option “--strandedness.” To improve the accuracy of the measurement, the “--estimate-rspd” option was applied. All other options were set to default values. To normalize sequencing depth among samples, FPKM and TPM values were calculated.

### Molecular subtyping and metagene selection

Molecular subtyping of the ovarian cancer patient group data was conducted using the TCGA-OV clustering method [18]. Genes were first quantile-normalized, and 4,579 genes were selected based on the median absolute deviation method with a threshold of 30. The R package NMF and the feature selection method [44] were used for clustering the patient groups and metagenes through NMF [40]. Pathway analysis of metagenes divided into individual NMF clusters was performed using the R package clusterProfiler [45] based on the HALLMARK, KEGG, Wikipathway, and REACTOME gene sets from the Molecular Signature Database (MsigDB) [46]. At least three pathways were annotated for each cluster, which contained the most metagenes and was under the adjusted p-value threshold (**P* *< 0.01).

### Gene correlation network analysis

A total, 1,949 RNAs were selected based on variance and correlation scores (correlation score > 0.55, Pearson’s method) and subjected to unsupervised hierarchical clustering using complete linkage. Ten pathway clusters were detected, and cluster characterization was conducted through enrichment analysis using hallmark gene sets from MSigDB. The protein network layout was created using the ForceAtlas2 algorithm from Gephi, a network visualization platform, balancing linear attraction and repulsion forces through the Barnes–Hut method with a scaling parameter set at 7.0, and gravity value of 1.0, while preventing overlapping nodes (K-core > 5).

### Immune deconvolution analysis

Ovarian cancer tissue samples were analyzed using the xCell algorithm [47] to computationally estimate the relative abundance of immune cell types. This involved preprocessing bulk tissue gene expression data to infer immune cell composition, providing detailed insights into the ovarian cancer microenvironment. The resulting immune cell scores were averaged using NMF clusters. Stromal and immune scores were measured using the ESTIMATE tool [48], which is a computational method used to infer the presence of stromal and immune cells in tumor samples based on gene expression data. The ESTIMATE score is an integrated form of stromal and immune scores that provides an overall indication of tumor purity.

### Cancer cell vulnerability and drug sensitivity analysis

We obtained CERES scores, which represent CRISPR gene essentiality, from the Cancer Dependency Map (DepMap), integrating CRISPR knockout screens from the Achilles Project with genomic data from the Cancer Cell Line Encyclopedia (CCLE). From a pool of 74 cell lines primarily associated with ovarian cancer, those with CERES scores below –0.5 were identified as vulnerable and dependent. To assess the significance of vulnerabilities across cancer types, we used the Wilcoxon rank-sum test (**P* *< 0.05).

Next, ovarian cancer cell lines underwent NMF clustering, similar to our cohort, resulting in the identification of four clusters, excluding a proliferative cluster but including one linked to the estrogen response. Subtype-specific gene dependencies were examined using the same thresholds.

### Cancer specific antigen analysis

Cancer germline antigen genes were compiled from a previous study [49]. Out of the 226 candidate cancer germline genes, outliers were additionally filtered based on a threshold exceeding three standard deviations from the mean. To enable a comparative analysis of the expression of these selected genes, tissue-specific transcripts per million (TPM) data from the Genotype-Tissue Expression (GTEx) project [50] were integrated and merged with the TPM data obtained from the transcriptomic data of the cohort.

### Establishment of survival prediction model

Metagene selection was conducted based on the Cox model using the R package “survival” [51] (**P* *< 0.05) and further filtered using logistic regression and backward elimination. The risk score was calculated by 1/(1 + exp(–regression coefficients x gene expression)), and was further used to construct the nomogram. The nomogram was generated using the “rms” R package. ROC analysis was performed to explore the prognostic performance.

## Supporting information

S1 Fig Effect of genomic alteration in immune score. (A) Boxplot describing no significant difference in tumor purity between TP53 mutated group against TP53 wild-type group. (B) Copy number alteration heatmap over 111 high grade serous ovarian cancer samples (x-axis) along chromosomal location (y-axis).
(TIF)

S2 Fig Estimating the factorization rank of NMF clustering and consensus map. (A) The figure showcases the outcomes of estimating the Non-Negative Matrix Factorization (NMF) factorization parameter “*k*” across a range from 2 to 6 on the 111 KNCC-HGSOV cohort. Quality measures are derived from 10 independent runs for each “r” value (left panel). On the right, the consensus map of the KNCC-HGSOV cohort is depicted for “r” values ranging from 2 to 6. (B) NMF factorization result parameter and consensus map of 44 neoadjuvant treated samples, (C) and on 79 treatment-naïve samples. Red boxes indicate the parameter of the selected *k* value that balances reconstruction accuracy (sparseness and dispersion), stability (cophenetic), and interpretability (silhouette).
(TIF)

S3 Fig Gene expression heatmap featured by NMF cluster of neoadjuvant treated and treatment-naïve samples. (A) The heatmap illustrates the features representing each of the 3 NMF clusters within the neoadjuvant treated cohort consisting of 44 samples. (B) In the treatment-naïve cohort comprising 79 samples, a total of 5 clusters were identified following NMF clustering.
(TIF)

S4 Fig Immune deconvolution and statistical analysis by 4 NMF clusters. (A) Computationally estimated immune cell component is visuallized on heatmap. (B) The boxplot illustrates the statistically different proportions of keratinocyte and skeletal muscle cells across 4 NMF clusters. (C) Boxplot depicting chemokine expression by 4 clusters.
(TIF)

S5 Fig Comparative analysis of CGA expression in KNCC OV cohort and normal cells. (A) Comparison of overall enrichment of the immune related pathways such as allograft rejection and interferon alpha response in 29 TCGA pan cancer subtype integrated with KNCC-OV cohort, highlighting when decomposed by TCGA cluster and 4 KNCC NMF cluster, respectively. (B) Linear regression comparing immune score and HRD score.
(TIF)

S6 Fig Feature of ovarian cancer compared to other cancer subtypes. (A) Boxplots exhibiting significantly higher dependency of ovarian cancer cells on TOR2A and integrin-linked kinase genes when knocked down. CERES score and cell line data were repurposed from the public DEPMAP and CCLE databases. (B) Ovarian cell lines from the CCLE database were classified into four subgroups using the NMF algorithm and were examined for their molecular-subtype specific gene dependency.
(TIF)

S7 Fig Comparative analysis of CGA expression in KNCC OV cohort and normal cells. (A) Violin plot comparing CGA expression in KNCC OV cohort against normal cell, data sourced from GTEx.
(TIF)

S8 Fig Comparative analysis of TP53 mutated and TP53 WT samples using transcriptome and copy number data. (A) Barplot that exhibits the significant transcriptomic difference in GSEA enrichment between TP53 mutated and TP53 WT samples. (B) Volcano plot that exhibits copy number difference between TP53 mutated and TP53 WT samples. Oncogenes are labeled with red color.
(TIF)

S1 Table Clinical information of 111 high-grade serous ovarian cancer samples. (XLSX)

S2 Table Copy number data of 111 high-grade serous ovarian cancer samples. (XLSX)

S3 Table Total of 2,790 genes with significant cis-effect and positive correlation (FDR < 0.05). (XLSX)

S4 Table NMF clustering result, total of 4,597 features and 111 samples matched with annotation. (XLSX)

S5 Table NMF clustering result of DepMap public cell lines subsetted with ovarian cancer. (XLSX)

S6 Table Result of DEG analysis comparing cancer-specific antigen overexpressed group and other. (XLSX)
