# Supplementary figures and images for: Integrative transcriptomic analysis of Korean high-grade serous ovarian cancer
Source: PLoS Genet. 2025 Sep 15;21(9):e1011660. doi: 10.1371/journal.pgen.1011660 (PMC12494275; doi:10.1371/journal.pgen.1011660)

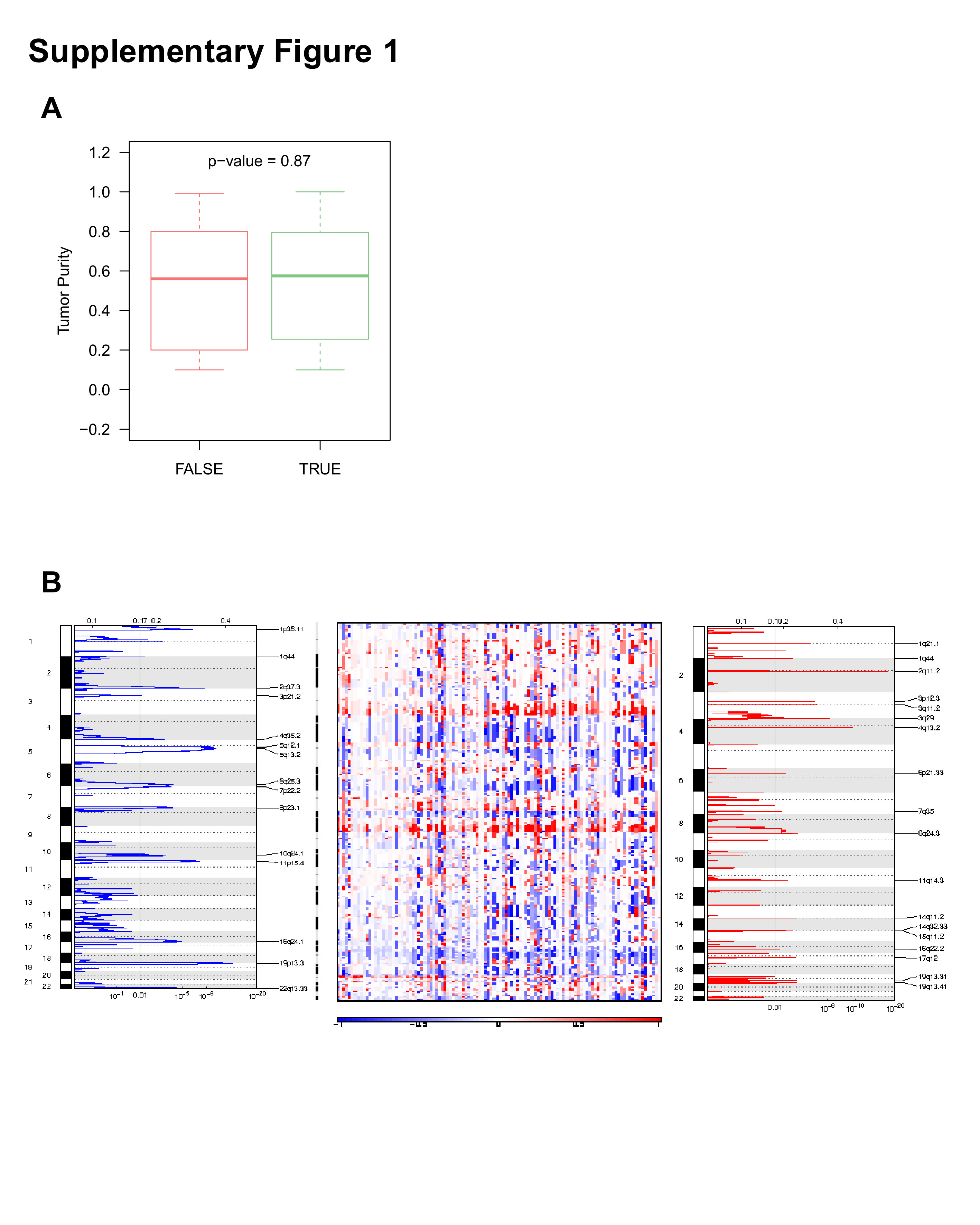

Supplement: S1 Fig — (A) Boxplot describing no significant difference in tumor purity between TP53 mutated group against TP53 wild-type group. (B) Copy number alteration heatmap over 111 high grade serous ovarian cancer samples (x-axis) along chromosomal location (y-axis). (TIF) [file pgen.1011660.s001.tif]

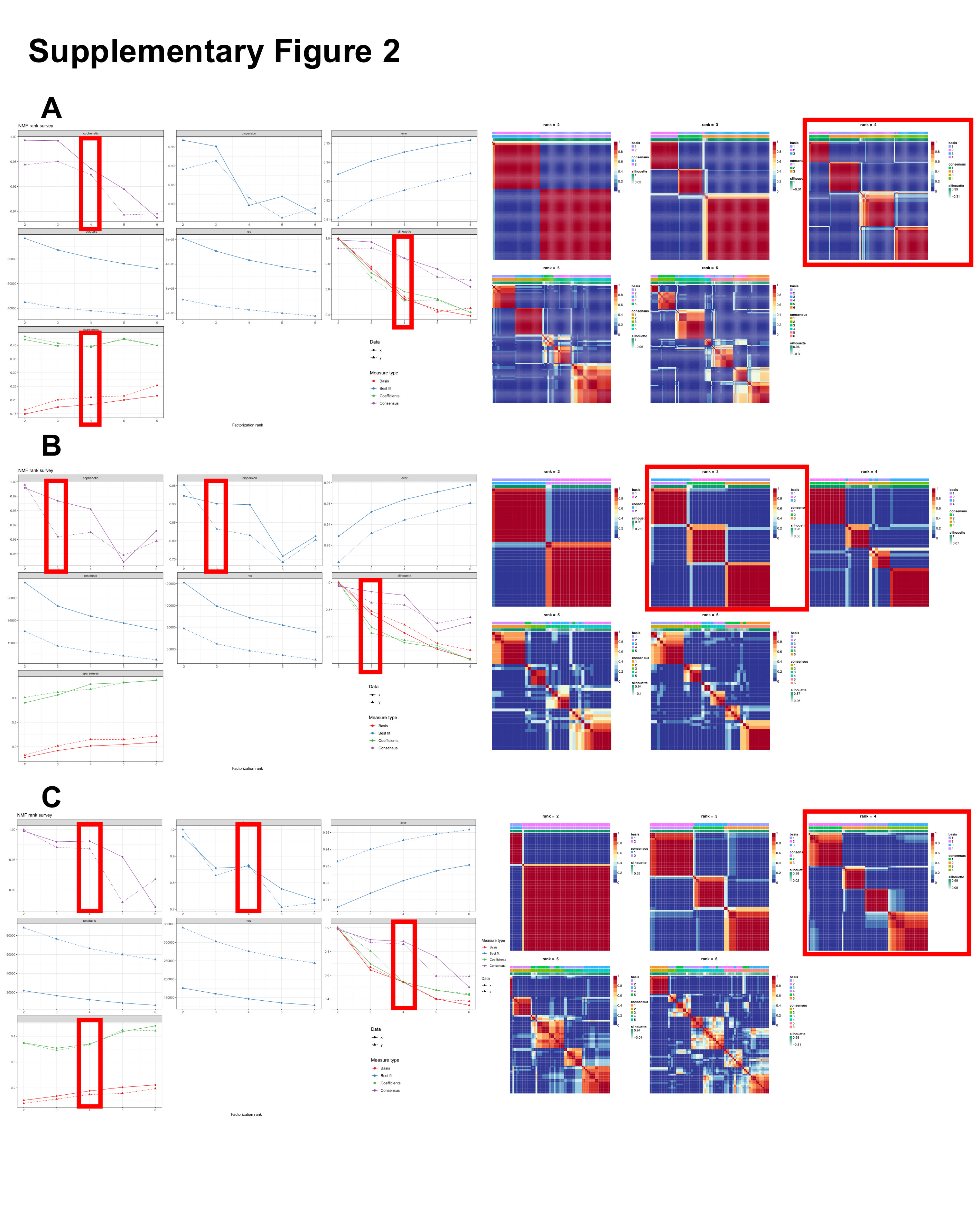

Supplement: S2 Fig — (A) The figure showcases the outcomes of estimating the Non-Negative Matrix Factorization (NMF) factorization parameter “k” across a range from 2 to 6 on the 111 KNCC-HGSOV cohort. Quality measures are derived from 10 independent runs for each “r” value (left panel). On the right, the consensus map of the KNCC-HGSOV cohort is depicted for “r” values ranging from 2 to 6. (B) NMF factorization result parameter and consensus map of 44 neoadjuvant treated samples, (C) and on 79 treatment-naïve samples. Red boxes indicate the parameter of the selected k value that balances reconstruction accuracy (sparseness and dispersion), stability (cophenetic), and interpretability (silhouette). (TIF) [file pgen.1011660.s002.tif]

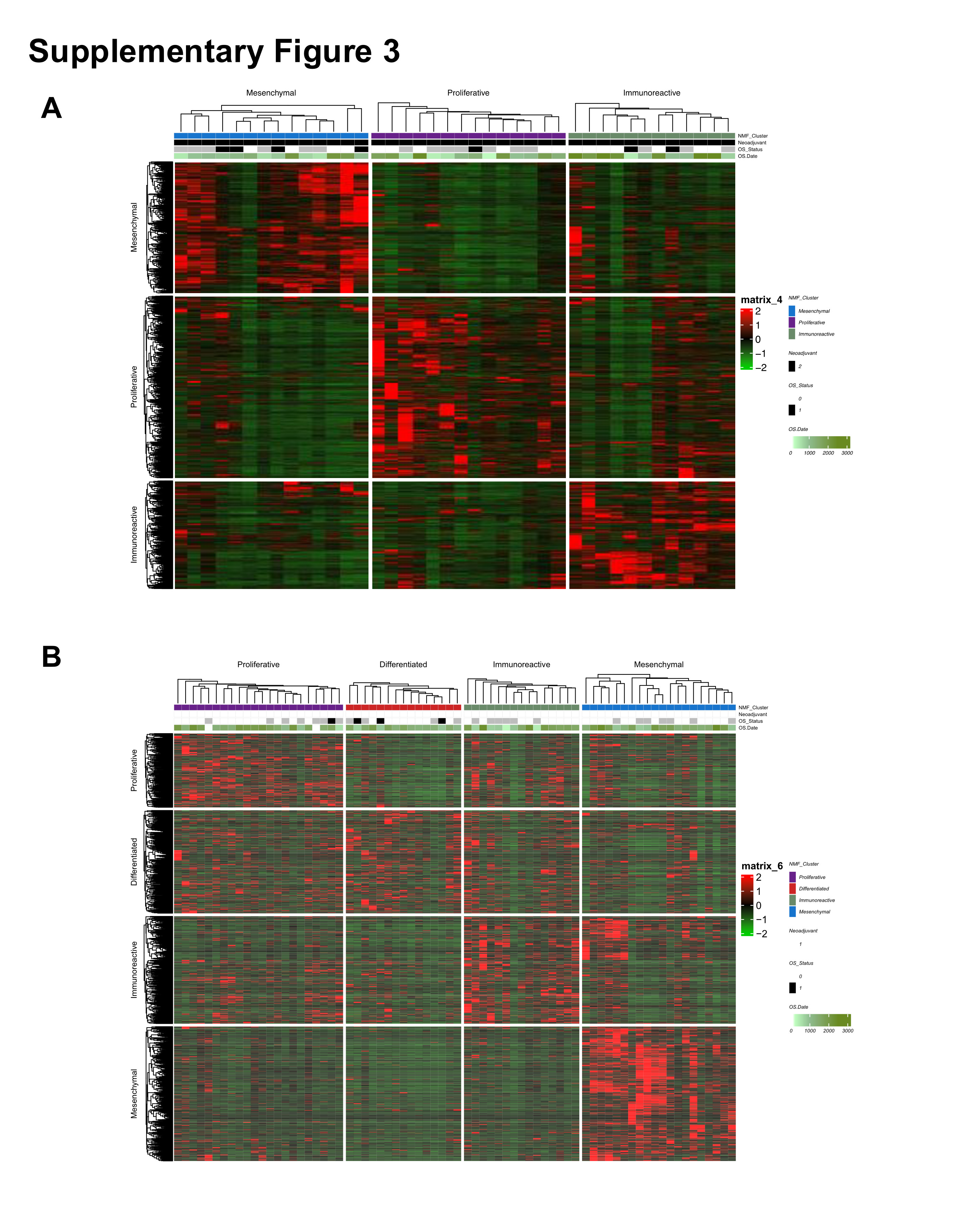

Supplement: S3 Fig — (A) The heatmap illustrates the features representing each of the 3 NMF clusters within the neoadjuvant treated cohort consisting of 44 samples. (B) In the treatment-naïve cohort comprising 79 samples, a total of 5 clusters were identified following NMF clustering. (TIF) [file pgen.1011660.s003.tif]

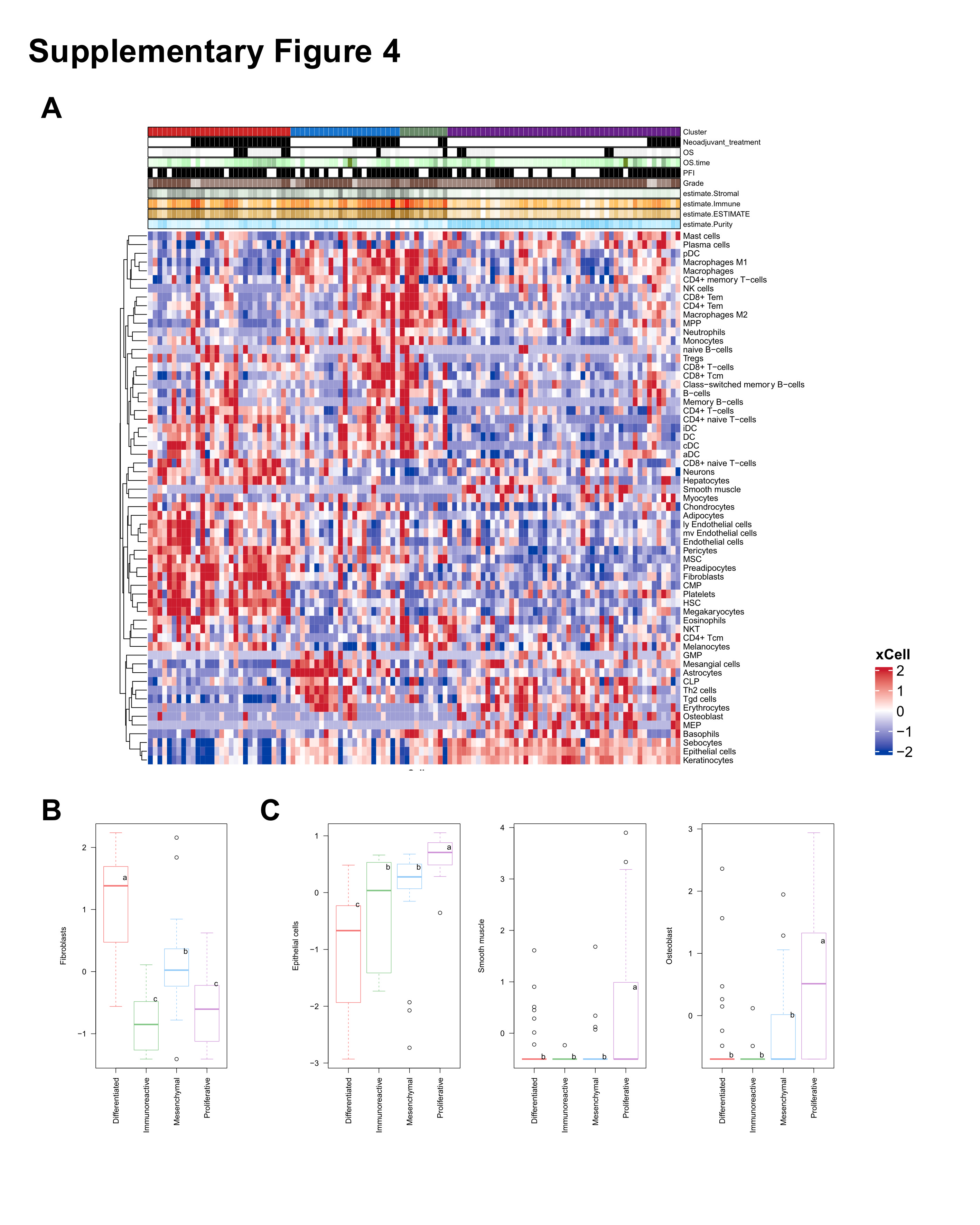

Supplement: S4 Fig — (A) Computationally estimated immune cell component is visuallized on heatmap. (B) The boxplot illustrates the statistically different proportions of keratinocyte and skeletal muscle cells across 4 NMF clusters. (C) Boxplot depicting chemokine expression by 4 clusters. (TIF) [file pgen.1011660.s004.tif]

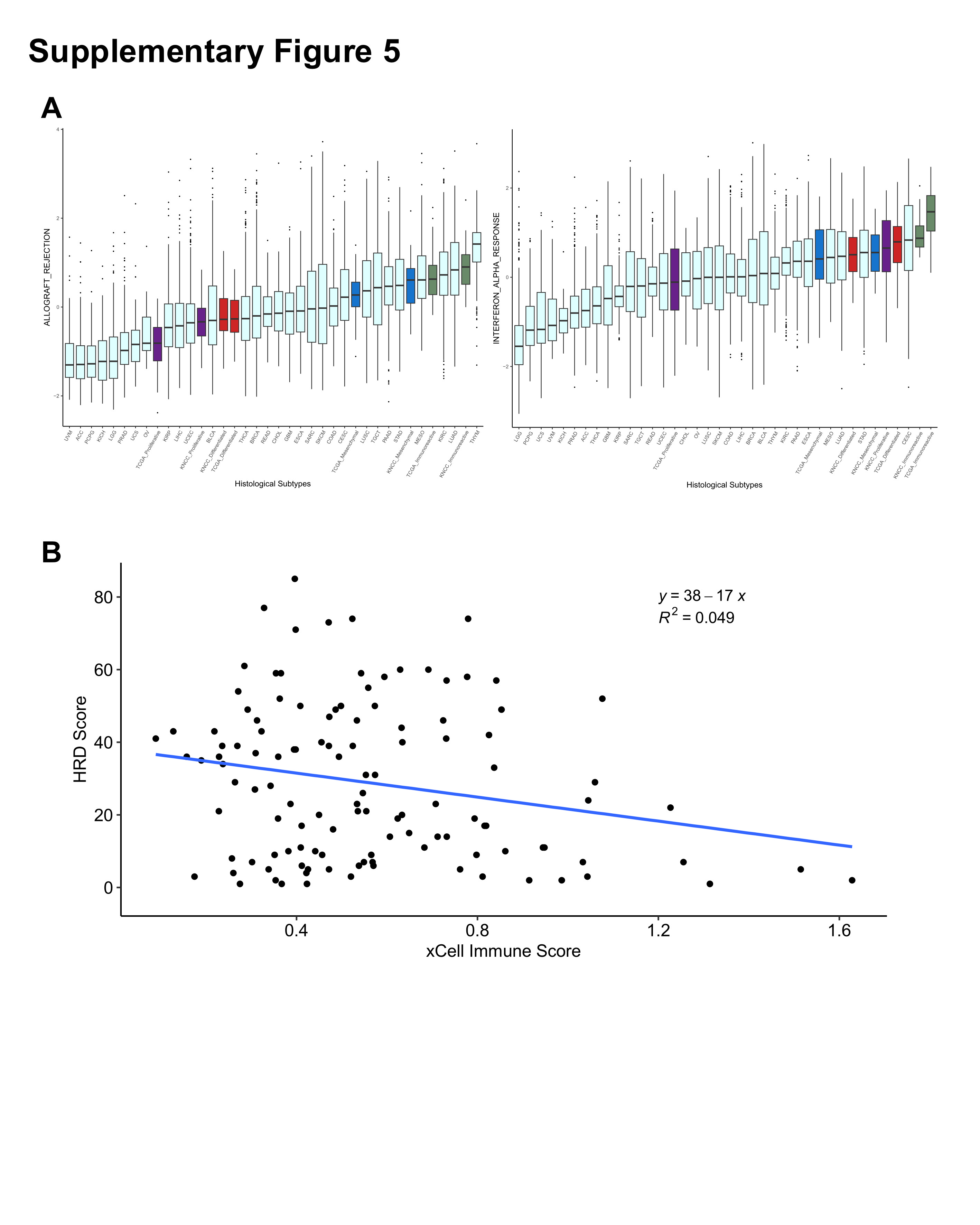

Supplement: S5 Fig — (A) Comparison of overall enrichment of the immune related pathways such as allograft rejection and interferon alpha response in 29 TCGA pan cancer subtype integrated with KNCC-OV cohort, highlighting when decomposed by TCGA cluster and 4 KNCC NMF cluster, respectively. (B) Linear regression comparing immune score and HRD score. (TIF) [file pgen.1011660.s005.tif]

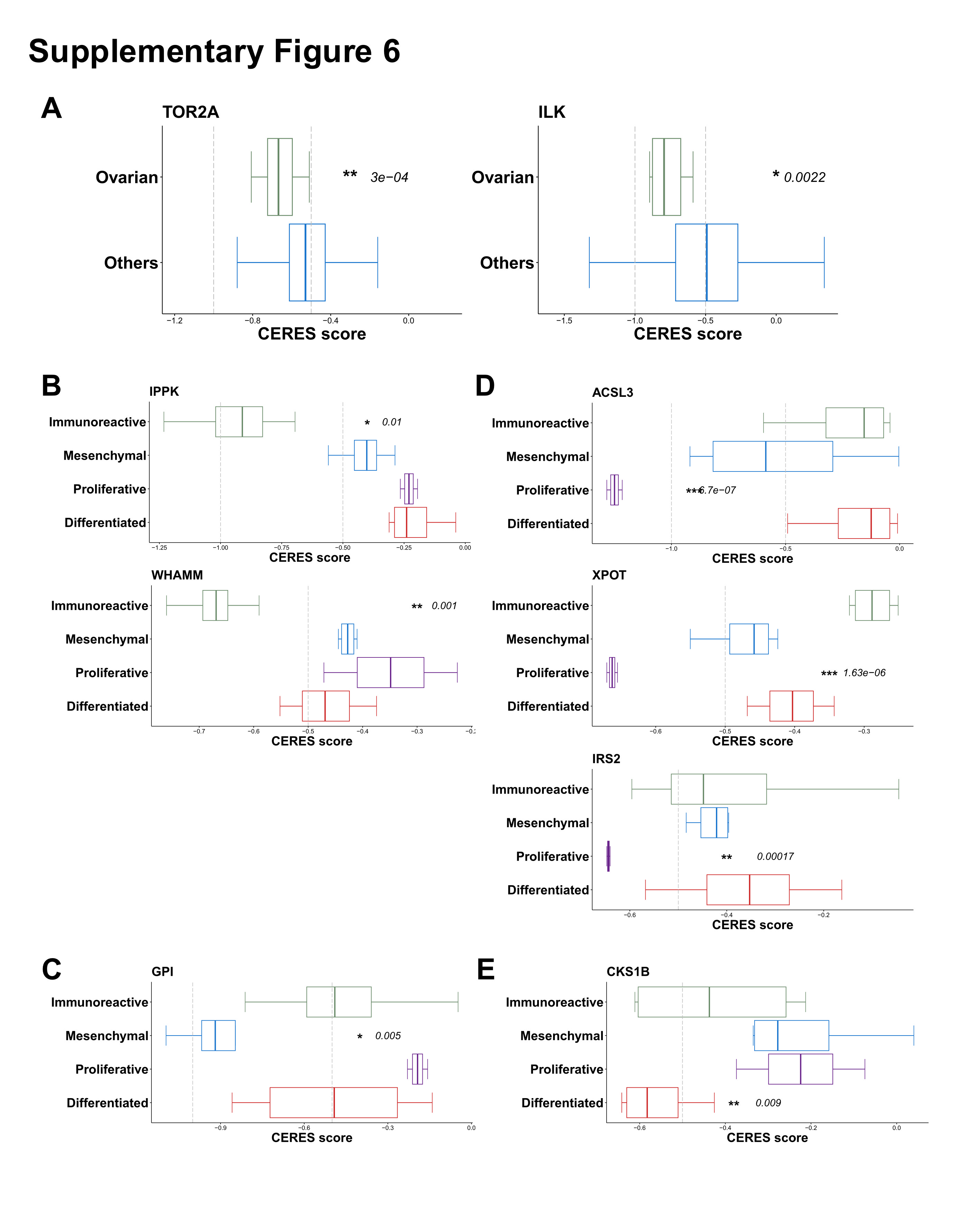

Supplement: S6 Fig — (A) Boxplots exhibiting significantly higher dependency of ovarian cancer cells on TOR2A and integrin-linked kinase genes when knocked down. CERES score and cell line data were repurposed from the public DEPMAP and CCLE databases. (B) Ovarian cell lines from the CCLE database were classified into four subgroups using the NMF algorithm and were examined for their molecular-subtype specific gene dependency. (TIF) [file pgen.1011660.s006.tif]

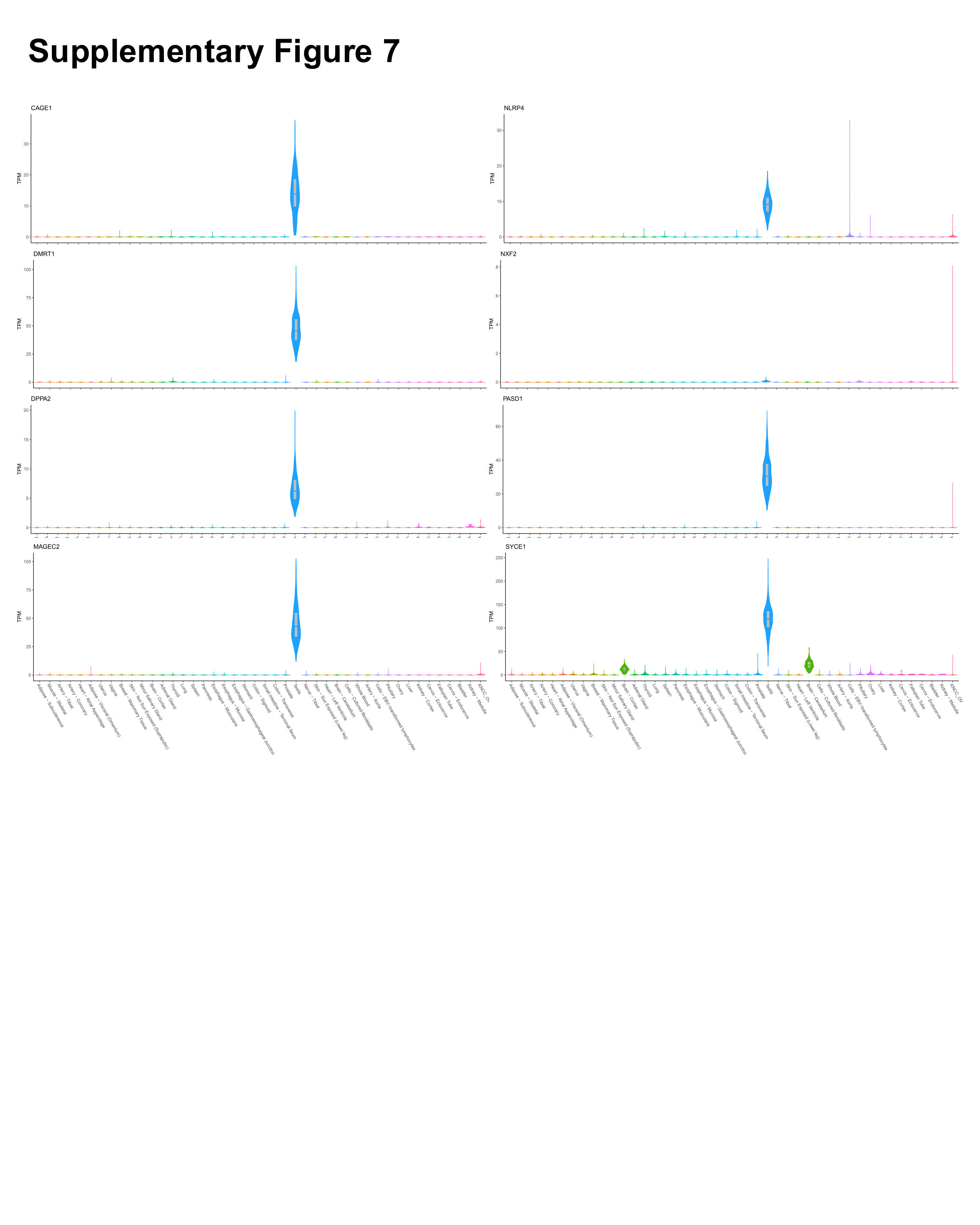

Supplement: S7 Fig — (A) Violin plot comparing CGA expression in KNCC OV cohort against normal cell, data sourced from GTEx. (TIF) [file pgen.1011660.s007.tif]

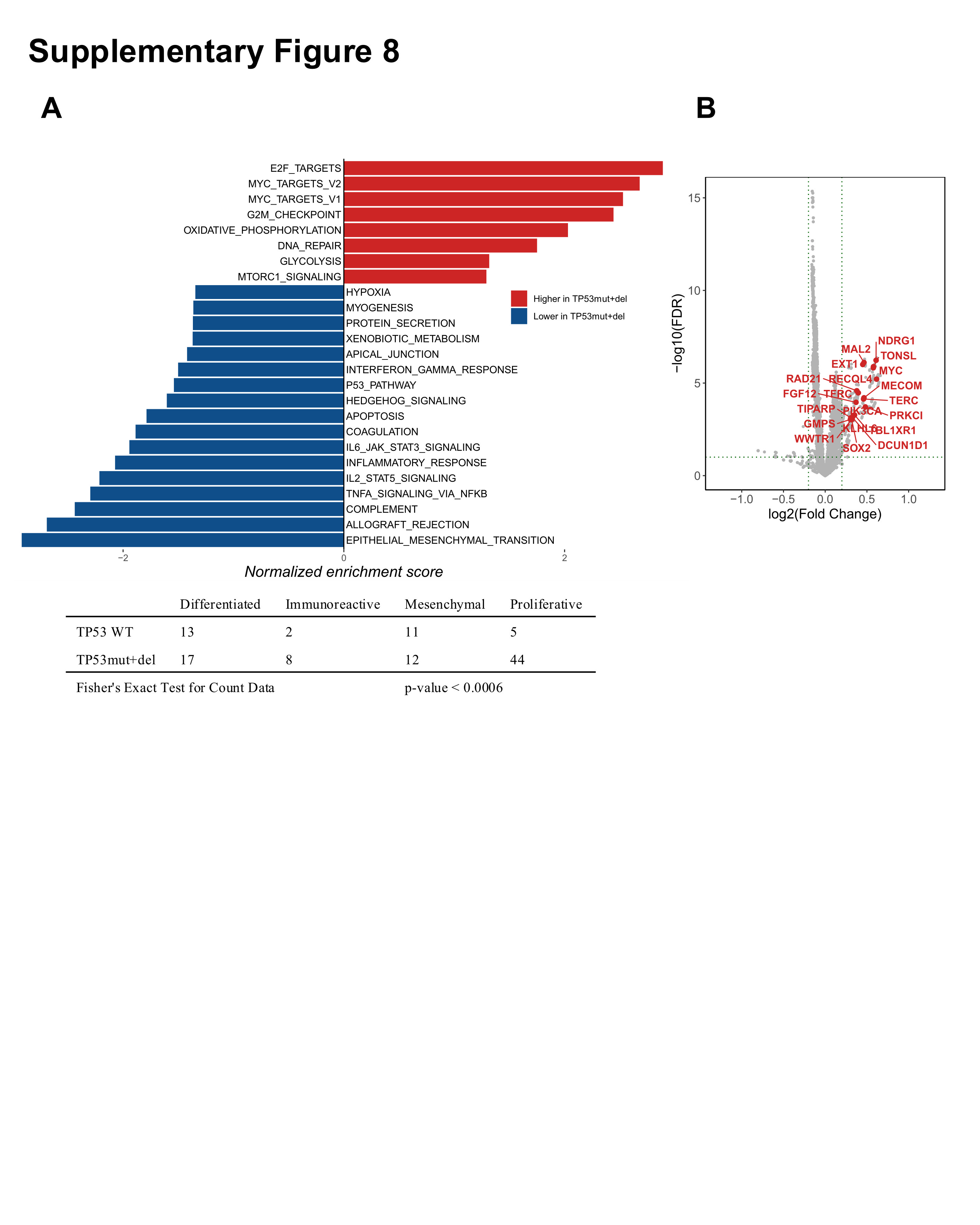

Supplement: S8 Fig — (A) Barplot that exhibits the significant transcriptomic difference in GSEA enrichment between TP53 mutated and TP53 WT samples. (B) Volcano plot that exhibits copy number difference between TP53 mutated and TP53 WT samples. Oncogenes are labeled with red color. (TIF) [file pgen.1011660.s008.tif]
